# Unraveling the functional dynamics of *Caenorhabditis elegans* stress‐responsive omega class GST‐44

**DOI:** 10.1111/febs.70088

**Published:** 2025-04-05

**Authors:** Charlotte Sophia Kaiser, Milena Lubisch, Emma Schröder, Luka Ressmann, Marie Nicolaus, Dustin Leusder, Sven Moyzio, Robert Peuss, Antonio Miranda‐Vizuete, Eva Liebau

**Affiliations:** ^1^ Institute of Integrative Cell Biology and Physiology University of Münster Germany; ^2^ Redox Homeostasis Group, Instituto de Biomedicina de Sevilla (IBIS) Hospital Universitario Virgen del Rocío/CSIC/Universidad de Sevilla Spain

**Keywords:** arsenic, *C. elegans*, glutathione transferase, stress response

## Abstract

Glutathione transferases from the omega class are notable for their roles in redox regulation and cellular stress response. In this study, we conducted a comprehensive functional characterization of GST‐44, an omega‐class glutathione S‐transferase (GSTO), in *Caenorhabditis elegans*, focusing on its role in cellular defense mechanisms against stress. Biochemical analysis revealed GSTO‐specific enzymatic activities of recombinant GST‐44, including dehydroascorbate reductase, thioltransferase, and arsenate reductase activities. Using transgenic GFP reporter strains, we identified predominant expression of GST‐44 in the intestine and excretory H‐cell, with significant upregulation observed under diverse stress conditions. Induction of GST‐44 was particularly pronounced in the intestine in response to pathogen‐, oxidative‐, and endoplasmic reticulum stress. Notably, under arsenic stress, the expression of *gst‐44* was significantly upregulated in the excretory system of the worm, underscoring its critical role in mediating arsenic detoxification. Moreover, we demonstrated the induction of GST‐44 using dimethyl fumarate, a highly specific mammalian Nrf‐2 activator. The upregulation of GST‐44 during arsenic stress was dependent not only on the oxidative stress response transcription factor SKN‐1/Nrf2 but also on PHA‐4. The deletion mutant strain *gst‐44(tm6133)* exhibited reduced stress resistance and a shortened lifespan, with a highly diminished survival rate under arsenic stress compared to other CRISPR‐generated *C. elegans* GSTO deletion mutants. Our findings highlight the essential role of GST‐44 in mediating arsenic detoxification, as well as in stress adaptation and defense mechanisms in *C. elegans.*

## Graphical abstract

Omega‐class glutathione S‐transferases (GSTs) are integral to redox regulation and cellular stress responses. This study characterized GST‐44 in *Caenorhabditis elegans*, revealing its predominant expression in the intestine and excretory H‐cell, with significant upregulation under various stress conditions. Particularly under arsenic stress, *gst‐44* expression is mainly induced in the excretory H‐cell and regulated by SKN‐1 and PHA‐4. Deletion of *gst‐44* impairs stress resistance and shortens lifespan, highlighting its critical role in detoxification.

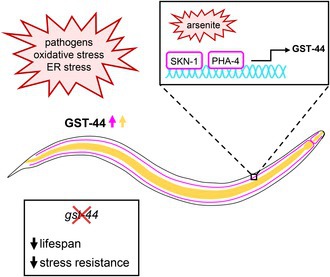

**Abbreviations**
CDNB: 1‐chloro‐2,4‐dinitrobenzene
DHAR: dehydroascorbate reductase
DMF: dimethylformamide
GPX: glutathione peroxidase
GSH: glutathione
GST: glutathione S‐transferase
KEAP1: Kelch‐like ECH‐associated protein 1
Nrf2: nuclear factor erythroid 2‐related factor 2
PHA‐4: defective pharynx development‐4
SKN‐1: skinhead‐1
TCEP: tris(2‐carboxyethyl)phosphine

## Introduction

The glutathione S‐transferase (GST) superfamily has evolved to include a wide variety of enzymes that serve multiple functions in cellular metabolism and provide protection against environmental stressors [1]. Primarily, GSTs act as detoxification enzymes by catalyzing the conjugation of glutathione (GSH) to electrophilic substances, facilitating their biotransformation and elimination from cells. Additionally, GSTs serve as ligands, participating in the intracellular transport and storage of hydrophobic ligands such as hormones or metabolites. Furthermore, some GSTs have been associated with the modulation of cellular signaling pathways, including those involved in stress responses and survival [1, 2, 3].

The dynamic evolution of the GST superfamily has resulted in a diverse range of enzymes with versatile functions, playing crucial roles in maintaining cellular homeostasis and facilitating adaptive responses. Within this superfamily, the omega class (GSTO) distinguishes itself from other GST classes by possessing a catalytic cysteine residue at the active site instead of a serine or tyrosine residue. This unique feature grants GSTO enzymes distinct catalytic properties, most significantly dehydroascorbate reductase (DHAR) and glutaredoxin‐like activities, distinguishing them from other GST classes [4]. Other enzymatic reactions include the reduction of S‐(phenacyl)glutathione to acetophenones and the reduction in monomethyl and dimethyl arsonate [5, 6, 7]. Recently, GSTOs have been shown to participate in the glutathionylation cycle of proteins [8] indicating their critical role in redox homeostasis [9].

GSTOs have been extensively investigated in numerous biologically and clinically relevant pathways and disorders, encompassing the modulation of calcium release channels [10], the posttranslational processing of the pro‐inflammatory cytokine interleukin‐1ß [11] or the regulation of lipopolysaccharide‐stimulated inflammatory responses in macrophages [12]. Recently, GSTO1 was shown to promote NLRP3 inflammosome activation in macrophages by deglutathionylating the central inflammosome adapter protein ASC [13]. Furthermore, polymorphic variants of GSTOs have been implicated in the age at onset and progression of neurodegenerative diseases such as Alzheimer's and Parkinson's disease, as well as various types of cancer [14, 15, 16].

The soil‐dwelling nematode *Caenorhabditis elegans* possesses five genes that encode GSTOs: *gsto‐1*, *gsto‐2*, *gsto‐3*, *C02D5.4*, and *gst‐44* [17]. Among these genes, only *gsto‐1* has been investigated to a certain extent. Here, overexpression and silencing of the enzyme have provided evidence linking *gsto‐1* to increased stress resistance, specifically within the intestine, where expression was shown to be associated with the intestinal GATA‐type transcription factor ELT‐2 [18]. Moreover, under transient hypoxia, the GSTO‐1 is upregulated via a pathway involving the mechanistic target of rapamycin (mTOR) and ELT‐2, leading to an increase in lifespan. Notably, the hypoxia‐induced extension of lifespan depends on the presence of GSTO‐1 [19].

In this study, we demonstrated that the recombinant GST‐44 exhibits peroxidase and thioltransferase activity and can directly reduce arsenate using reduced glutathione as the electron donor. We explored the role of GST‐44 in the organism's stress response, particularly under arsenic exposure. Using a transgenic reporter strain, we analyzed its spatial and temporal expression patterns, uncovering significant variations depending on the stress type. Notably, GST‐44 expression was markedly upregulated under arsenic stress, primarily regulated by key transcription factors SKN‐1/Nrf2 and PHA‐4. Moreover, GST‐44 deletion mutants exhibited a shortened lifespan and reduced stress resistance when exposed to arsenic, oxidative, and endoplasmic reticulum stress. Our findings underscore the critical role of GST‐44 in mediating arsenic detoxification and in the organism's overall stress adaptation and defense mechanisms in *C. elegans*.

## Results

### Bioinformatic analysis

**Fig. 1:**
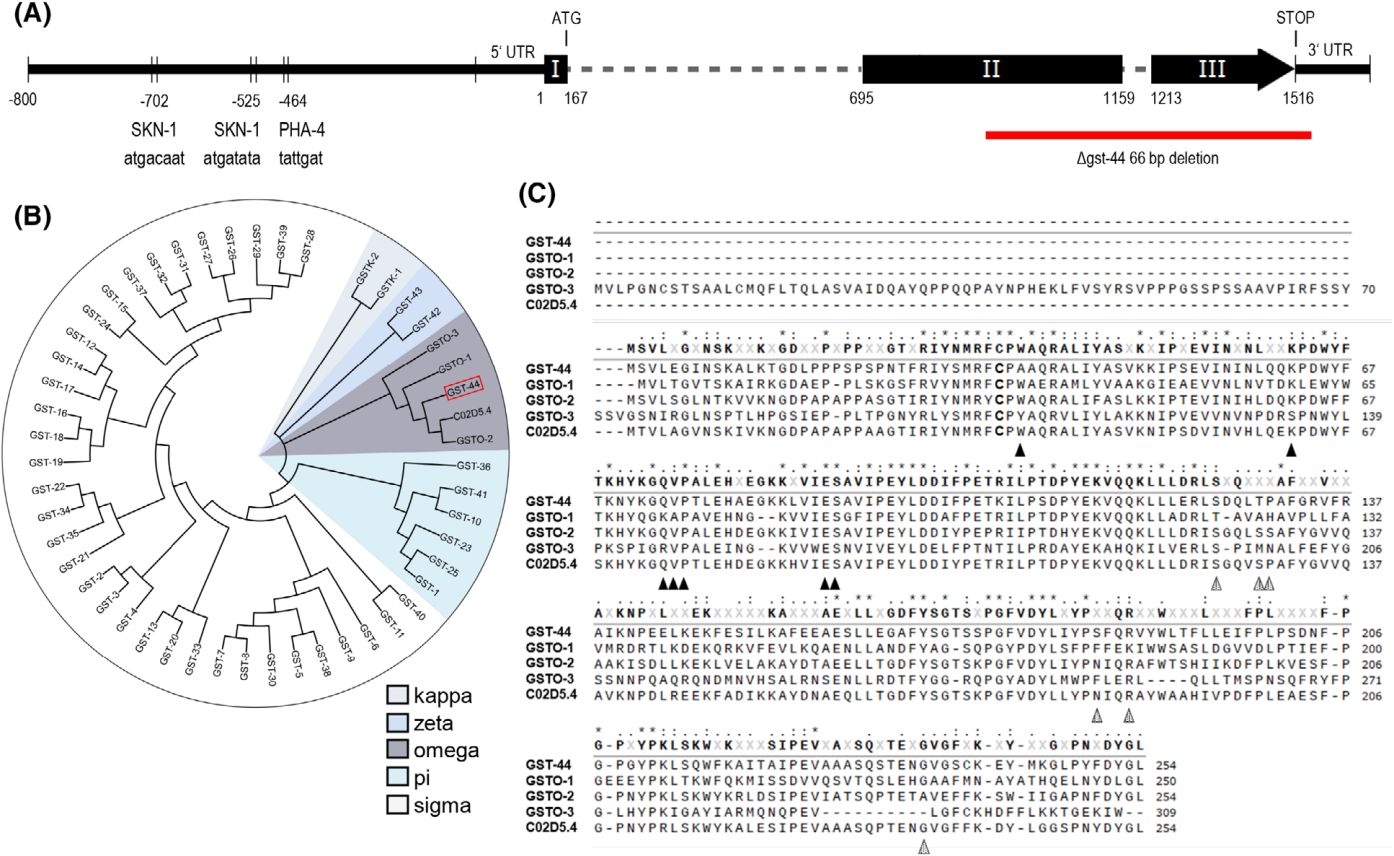
Bioinformatical analysis of *gst‐44*. (A) Depiction of the potential promoter region and gene structure of *gst‐44* on chromosome V. Black boxes indicate exons. The illustrated region shows the promoter region 800 base pairs upstream of the *gst‐44*, highlighting the binding regions for SKN‐1 and PHA‐4 and the 5′ and 3′ untranslated regions. The red line shows the position of the deletion found in *Δgst‐44*. (B) Optimal tree of GSTs in *C. elegans* using the minimal evolution method and the neighbor‐joining algorithm. The red box marks the GST‐44 within the family of the omega class GSTs. Evolutionary analyses were conducted in MEGA X. (C) Amino acid sequence alignment of the five GSTOs found in *C. elegans* (UniProt accession: GSTO‐1: P34345, GSTO‐2: P34277, GSTO‐3: O17234, GST‐44: O45352, C02D5.4: D7SFI3). The active site cysteine (gray shadow), conserved or conservatively substituted residues of the glutathione binding site (black triangle) and the hydrophobic substrate binding site (lined triangle) are shown. Alignment was generated using the program SnapGene (“*” 100%, “:” 63–99%, “.” 38–62% conservation).

The promoter region of the *gst‐44* revealed various transcription factor binding sites. Given that the omega class is known to be involved in stress response mechanisms, we specifically looked at binding sites for stress‐related transcription factors. Here, we identified two binding sites for SKN‐1 (located at positions −702 and −525) and PHA‐4 (positioned at −464), both of which play key roles in stress responsive processes (Fig. 1A).

The alignment and construction of a neighbor‐joining tree (Fig. 1B,C), based on the protein sequences of all GST from *C. elegans*, clearly demonstrate that GST‐44 belongs to the omega class. Furthermore, it is most closely related to GSTO‐1 and GSTO‐2.

The protein alignment of the five GSTOs from *C. elegans* (Fig. 1C) illustrates that the glutathione‐binding site (G‐site), situated within the N‐terminal domain, is composed of a set of remarkably conserved amino acid residues, which notably include the active site cysteine. In contrast, the hydrophobic substrate binding site (H‐site) found in the C‐terminal domain exhibits considerably more variability, permitting the accommodation of a wide spectrum of electrophiles.

### *Caenorhabditis elegans* GST‐44 enzymatic characterization

**Fig. 2:**
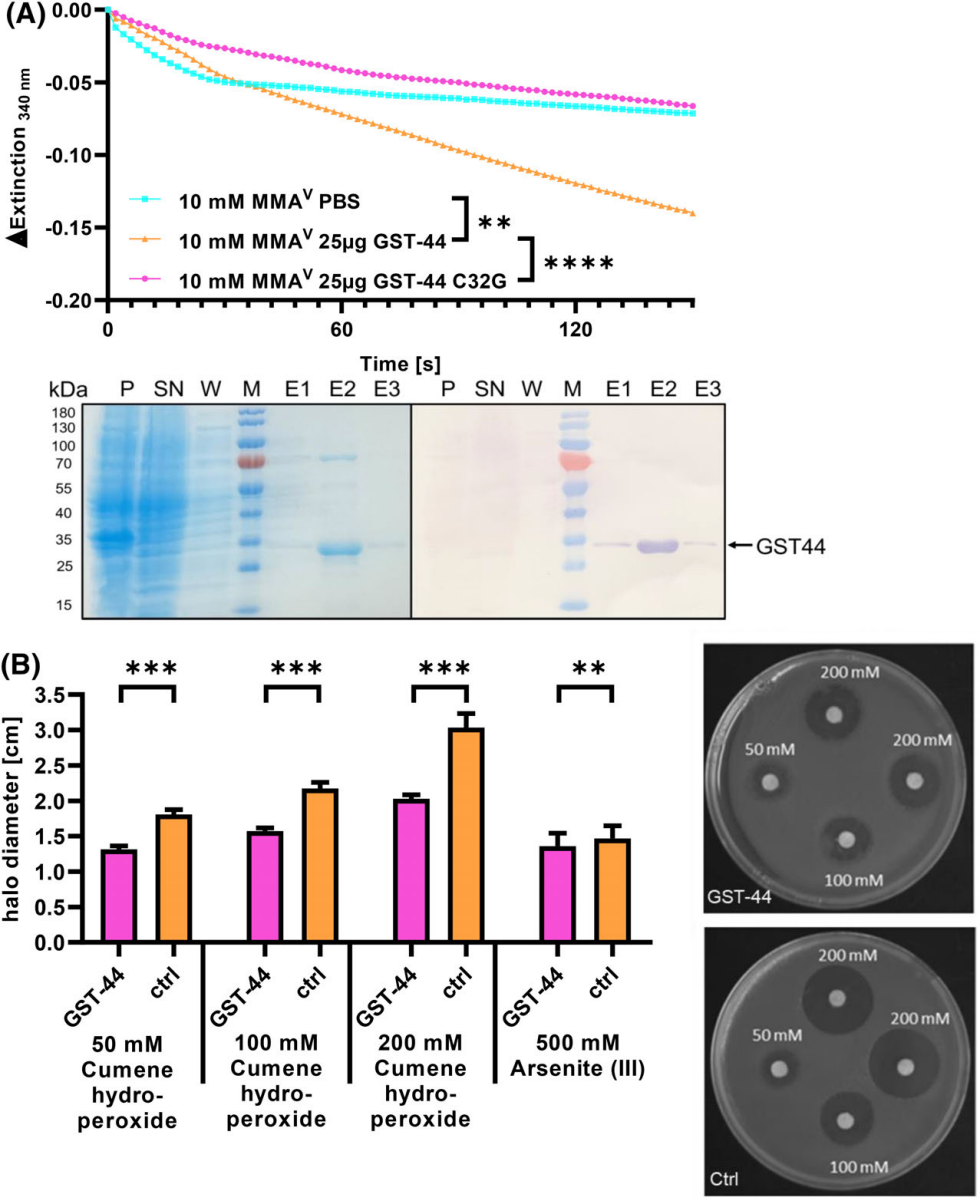
Enzyme activities of the GST‐44. (A) Spectrophotometric analysis of arsenate reductase activity. The decrease in NADPH absorbance at 340 nm indicates enzyme activity over a three minute period. Significant differences were calculated using the Student's *t*‐test as well and are marked with ** ≤ 0.01 and **** for *P* ≤ 0.0001 (*n* = 3). Representative purification of the recombinant GST‐44. Aliquots taken at each step of the purification process were analyzed on a Coomassie‐stained 12.5% SDS gel (left). Lane 1 (P, pellet); lane 2 (SN, supernatant); lane 3 (W, wash fraction); lane 4 (M, marker); lane 5–7 (E1‐3, elution fractions) and Western blot (right) analysis of the same fractions using an anti‐his antibody. (B) Disk diffusion assay using cumene hydroperoxide as stressor. *E. coli* expressing GST‐44 or the empty pJC40 vector were exposed to different concentrations (50, 100 and 200 mm) of cumene hydroperoxide and to As^III^ (500 mm). After overnight incubation, halo diameters of the inhibition zones around the filter disks were measured. Mean sizes of inhibition zones and standard errors are shown (left panel). Significant differences were calculated using the Student's *t*‐test and are marked with ** ≤ 0.01 and *** for *P* ≤ 0.001 (GST‐44 *n* = 21, CTRL *n* = 11). Representative disk diffusion assays with GST‐44 (top) and empty vector pJC40 as control (bottom) at indicated concentrations of cumene hydroperoxide (right panel).

After expression in *Escherichia coli*, the enzyme was successfully purified using NiNTA‐agarose and visualized through SDS/PAGE and western blot analysis. The bands observed in the elution fractions showed an approximate size of 30 kDa, closely aligning with the calculated size of 28.7 kDa for GST‐44, with an additional 1 kDa attributed to the His‐tag (Fig. 2A).

To evaluate the ability of the GST‐44 to protect against environmental stressors, bacteria expressing GST‐44 were exposed to different concentrations of cumene hydroperoxide (50, 100, 200 mm) and arsenite (As^III^) (100, 200, 500 mm). After overnight incubation, the inhibition zones around the stressor‐soaked filter disks were measured. A dose‐dependent reduction of the halos could be shown that started at 27% for 50 and 100 mm up to 33% for 200 mm of cumene hydroperoxide (Fig. 2B). In the presence of As^III^, only a small reduction in the halo size was observed. However, the decreased colony density made accurate measurements challenging. The native arsenic resistance mechanism in *E. coli*, encoded on an operon containing the genes *arsRDABC* [20], was considered in the experimental design, though its impact on the suitability of this test approach remains a point of consideration.

To further investigate the enzymatic properties of the GST‐44, various activity assays were performed. For the typical GST substrates 1‐chloro‐2,4‐dinitrobenzene (CDNB) and ethacrynic acid, no GSH‐conjugating activity could be shown (data not shown). However, since the disk diffusion assay indicated a protective effect against cumene hydroperoxide, we assessed the peroxidase activity of the recombinant protein and found a specific activity (0.37 ± 0.11 μmol·min^−1^·mg^−1^). Given the presence of a cysteine residue in the active site, we investigated thioltransferase activity using the artificial substrate 2‐hydroxyethyl disulfide (HEDS). We observed specific thioltransferase activity of 5.08 ± 0.2 μmol·min^−1^·mg^−1^ and a *K*_M_ of 0.16 mm. Furthermore, the enzyme was capable of using GSH as an electron donor to reduce dehydroascorbate (DHA) (6.35 ± 0.15 μmol·min^−1^·mg^−1^) and monomethylarsenate (MMA^v^) (0.635 ± 0.13 μmol·min^−1^·mg^−1^).

### GST‐44 expression under normal and stress conditions

**Fig. 3:**
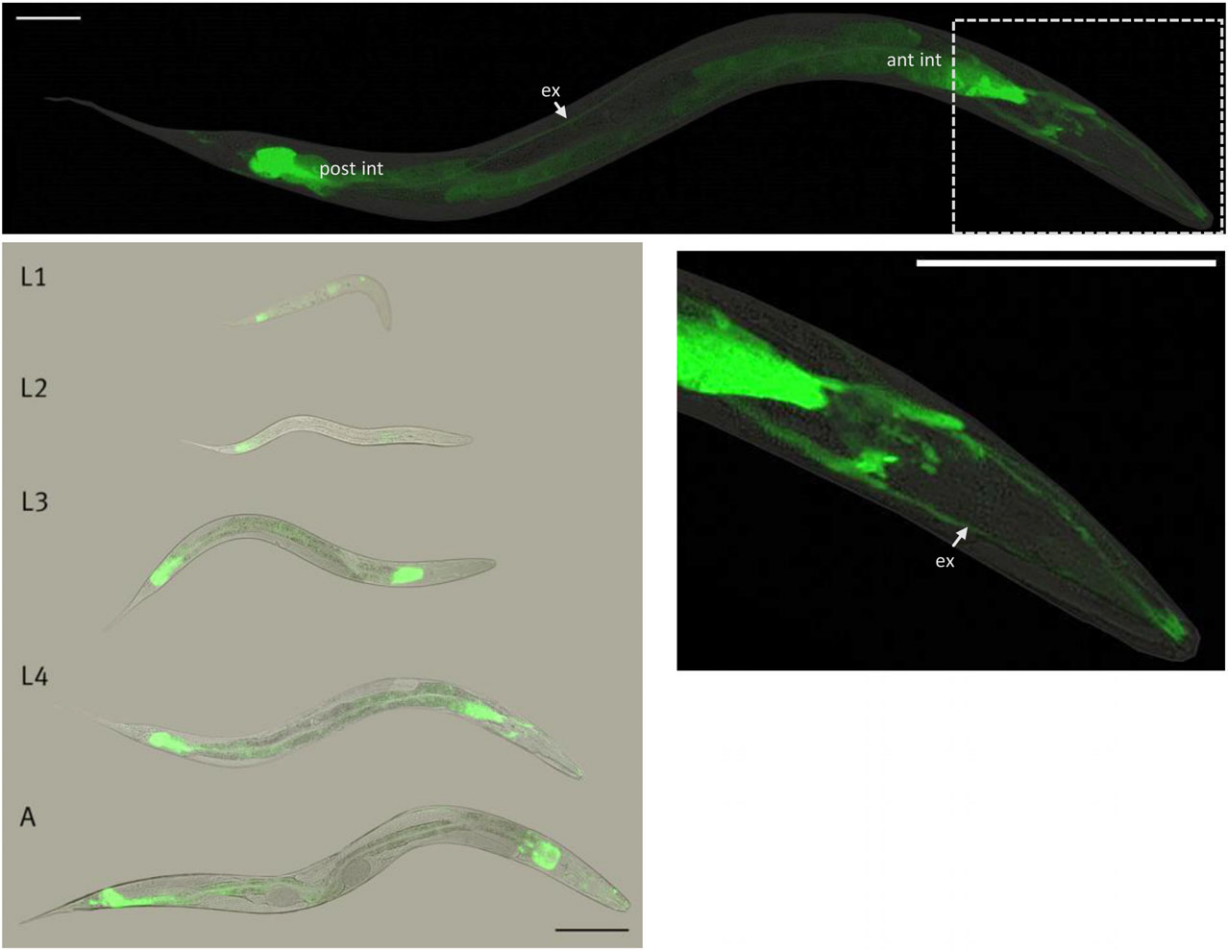
Analysis of the expression pattern of the translational reporter strain OH2204 (*gst‐44p::gst‐44::GFP + rol‐6*) under normal conditions. In adult worms (upper panel), the GST‐44::GFP fusion protein is predominantly expressed in the cytoplasm and nuclei of the intestine and also in the H‐shaped excretory cell (shown in detail). Fluorescence signals are detectable from the L1 stage through adulthood (lower panel). The expression is evident in both the anterior and posterior regions of the intestine. From the L3 stage onwards, expression in the excretory system is observed. The scale bar corresponds to 100 μm.

Using a reporter strain carrying the *gst‐44* promoter and gene fused to *gfp*, the expression of GST‐44 was observed (Fig. 3). Expression first becomes evident at the L1 stage, displaying a faint signal primarily in the intestine. As the worm progresses to the L3 stage, the signal intensifies and also becomes evident in the excretory system. In adult worms, a robust signal is observed in the cytoplasm of the anterior and posterior intestinal cells, accompanied by a weaker signal in the intestinal nuclei and in the excretory canal cell.

**Fig. 4:**
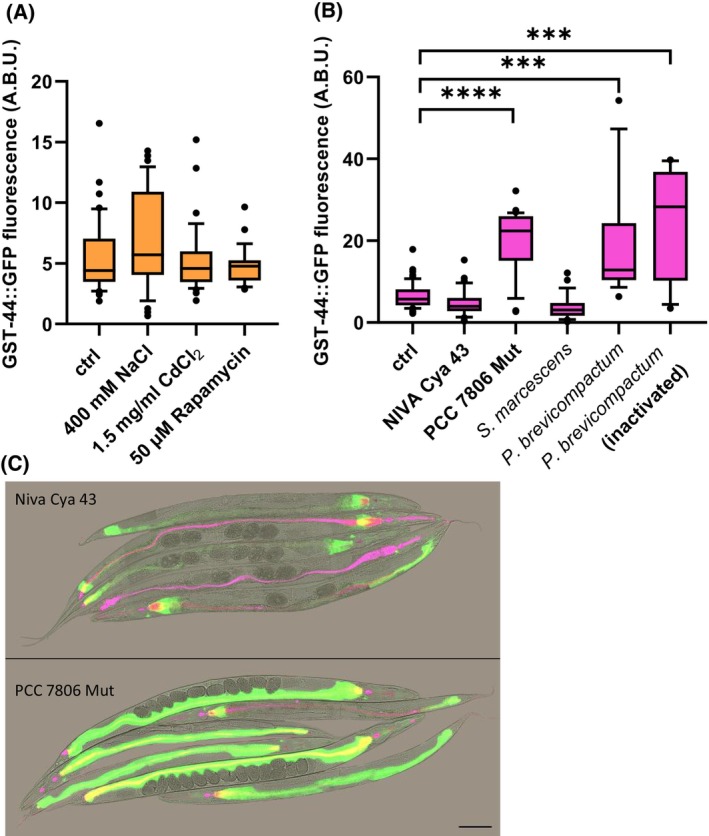
Induction of the GST‐44 under biotic and abiotic stress conditions. The transcriptional reporter strain OH2204 was used to assess the induction of GST‐44 in response to various stress conditions. Assays were conducted using stages L4 or young adults, and GFP signals were quantified using ImageJ. (A) Induction in response to biotic stresses, including 400 mm NaCl (4 h), 1.5 mg·mL^−1^ CdCl_2_ (4 h) and 50 μm rapamycin (4 h) (*n* = 30). Values are means from three independent experiments, and statistical significances were calculated with Anova on ranks using the Kruskal‐Wallis test. No statistical significances were detected. (B) Induction in response to biotic stresses, including exposure to two cyanobacteria strains (NIVA Cya 43 and PCC 7806 Mut), S. *marcescens*, *P. brevicompactum*, and heat inactivated *P. brevicompactum* (*n* = 25). Values are means from three independent experiments, and statistical significances were calculated with Anova on ranks using the Kruskal‐Wallis test with *** for *P* ≤ 0.001 and **** for *P* ≤ 0.0001. (C) Representative image of GST‐44 upregulation during pathogenic stress. Following a 5 h incubation on NGM plates supplemented with *M. aeruginosa* Niva Cya 43 (top) or PCC 7806 Mut (bottom), GFP signals were determined via laser scanning microscopy. Scale bar: 100 μm.

While osmotic stress, cadmium exposure, or treatment with the mTOR inhibitor rapamycin did not result in upregulation of GST‐44 expression (Fig. 4A), an increase in GFP fluorescence was detected following exposure to the ecologically relevant mold *Penicillium brevicompactum*, even when heat‐inactivated. Additionally, GST‐44 expression was upregulated specifically in response to the cyanobacteria strain PCC 7806 Mut, which contains a trypsin inhibitor, but not in NIVA Cya 43, which predominantly contains chymotrypsin inhibitors (Fig. 4 C). No induction of GST‐44 was observed following exposure to *Serratia marcescens* (Fig. 4B).

**Fig. 5:**
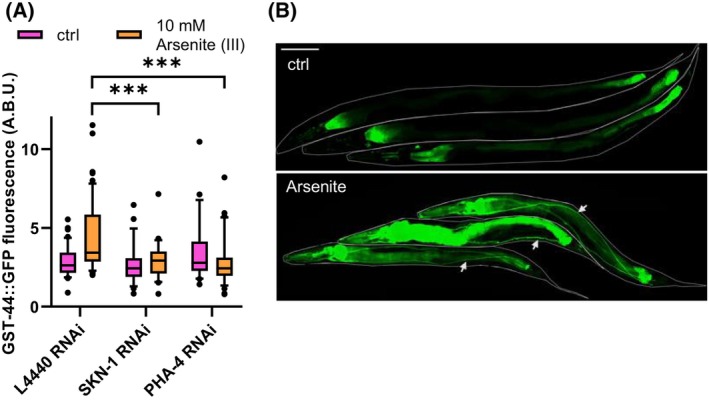
Expression of GST‐44::GFP in response to arsenic stress and under the knockdown of the transcription factors PHA‐4 and SKN‐1. (A) GST‐44 expression shows significant upregulation in response to 4 h exposure to 10 mm arsenic stress. The knockdown of the transcription factors PHA‐4 and SKN‐1 notably reduces expression under arsenic stress compared to worms without knockdown. (B) Representative image of *C. elegans* expressing GST‐44::GFP at basal levels. After incubation with 10 mm As^III^ for 4 h, GFP levels showed a dramatic increase, with significant upregulation also observed in the excretory organ (white arrows). Values are means from three independent experiments and statistical differences were calculated with Anova on ranks using the Kruskal‐Wallis test with *** for *P* ≤ 0.001 (control (ctrl) L4440 *n* ≥ 45, 10 mm As L4440 *n* ≥ 53, ctrl PHA‐4 *n* ≥ 22, ctrl SKN‐1 *n* ≥ 25, As PHA‐4 *n* ≥ 32, As SKN‐1 *n* ≥ 27).

In order to assess the involvement of the GST‐44 in response to arsenic stress, we exposed the GFP reporter worms to this stressor. Following a 4h incubation with 10 mm arsenic, we observed a significant increase in the fluorescence of the GST‐44::GFP reporter strain (Fig. 5A). This increase was noted in the intestine; however, a more prominent effect was observed in the excretory canal cell and pharyngeal gland cells (Fig. 5B).

To evaluate arsenic stress sensing, GST‐44::GFP transgenic dauer larvae were exposed to arsenite. The dauer stage was chosen because its closed intestine and active excretory system, responsible for balancing fluids and expelling waste, provide a means to differentiate between stress sensing through oral intake or cuticular leakage. However, no induction of *gst‐44* was observed in either the gut or excretory canal compared to controls (data not shown), preventing clear conclusions about the stress sensing route and suggesting that factors such as limited arsenite uptake, developmental suppression of *gst‐44*, or the activation of alternative detoxification pathways might be involved.

Two SKN‐1 and one PHA‐4 binding site, among others, were identified in the promoter region of *gst‐44* (Fig. 1). To explore the role of the transcription factors SKN‐1 and PHA‐4 in regulating *gst‐44* under arsenic stress, RNA interference (RNAi) experiments were conducted (Fig. 5A). In control experiments, the fluorescence of untreated GST‐44::GFP worms remained unchanged, irrespective of the RNAi bacteria they were exposed to. However, following a 4‐h incubation with 10 mm arsenic and RNAi targeting SKN‐1, there was a significant decrease in fluorescence compared with stressed control worms carrying the empty L4440 vector. Notably, RNAi targeting PHA‐4 also resulted in a substantial reduction in fluorescence. Our results indicate that the regulation of GST‐44 is not solely dictated by the SKN‐1 transcription factor in the presence of arsenic.

To ascertain whether SKN‐1 independently induces the expression of GST‐44 or if activation necessitates crosstalk between various transcription factors, dimethyl fumarate (DMF), a well‐known activator of NRF2/Foxo, was employed [21, 22].

**Fig. 6:**
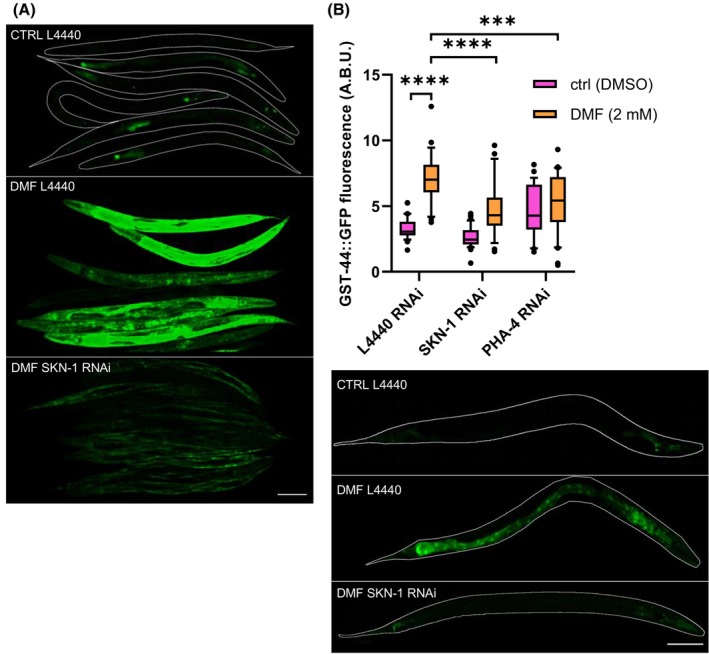
Expression of GST‐4::GFP and GST‐44::GFP in response to DMF and under the knockdown of the transcription factors PHA‐4 and SKN‐1. (A) Activation of GST‐4::GFP by DMF via SKN‐1. Upper: untreated control. Middle: Treatment with DMF. Bottom: Treatment with DMF and SKN‐1 knockdown. (B) GST‐44::GFP expression increases significantly after 18 h incubation with 2 mm DMF. Expression is significantly lower using the same treatment but performing an additional SKN‐1 knockdown. However, it is still higher in comparison to untreated worms where an SKN‐1 knockdown was performed. Under PHA‐4 knockdown, an increase in GST‐44::GFP expression is already observed in the untreated worms. Upon DMF treatment, a small reduction is observed in PHA‐4 RNAi worms in comparison to control worms. Statistical significances were calculated with Anova on ranks using the Kruskal‐Wallis test with *** for *P* ≤ 0.001, **** for *P* ≤ 0.0001. Control worms (ctrl) were treated with DMSO only (ctrl L4440 *n* = 34, ctrl SKN‐1 *n* = 43, DMF L4440 *n* = 26, DMF SKN‐1 *n* = 25). A.B.U., arbitrary brightness unit. Representative pictures of GST‐44 expression (upper: untreated control middle: 18 h treatment with 2 mm DMF bottom: 18 h treatment with 2 mm DMF and knockdown of SKN‐1). Scale bars correspond to 100 μm.

Therefore, our initial objective was to determine whether DMF exerts a similar impact on SKN‐1 in *C. elegans*. To achieve this, we utilized two reporter strains, LD1008 (*ldEx9 [skn‐‐(operon)::gfp + rol‐6(su1006)]*) and CL2166 (*dvIs19 [gst‐4p::GFP::NLS]*), commonly utilized as a proxy for SKN‐1 activation [23, 24]. Upon DMF treatment, a distinct increase in the fluorescence signal was evident in the LD1008 strain, extending beyond the two nuclei observed in the untreated control and present throughout the entire worm. In the strain CL2166, a robust activation of GST‐4 transcription was observed, but this activation was absent when the worms were additionally subjected to SKN‐1 RNAi (Fig. 6A).

After incubating with 2 mm DMF for 18 h, the fluorescence signal of GST‐44::GFP notably increased. However, this increase was significantly lower when SKN‐1 expression was suppressed via RNAi (Fig. 6B).

RNAi targeting *PHA‐4* led to an increase in GST‐44::GFP fluorescence compared with the untreated control. When treated with DMF, there was a slight reduction in fluorescence, but this effect was relatively minor compared with the reduction observed with RNAi targeting SKN‐1 (Fig. 6B).

The first intron of *gst‐44* is a long region with potential regulatory functions and harbors the short antisense transcript *F13A7.15*. While we did not identify additional SKN‐1 or PHA‐4 binding sites in this intron using WormBase, it remains plausible that this region contains regulatory elements influencing *gst‐44* expression. Future studies using CRISPR‐ or reporter gene‐based approaches to delete or modify the intron, including *F13A7.15*, could clarify its functional role in *gst‐44* regulation and stress response.

**Fig. 7:**
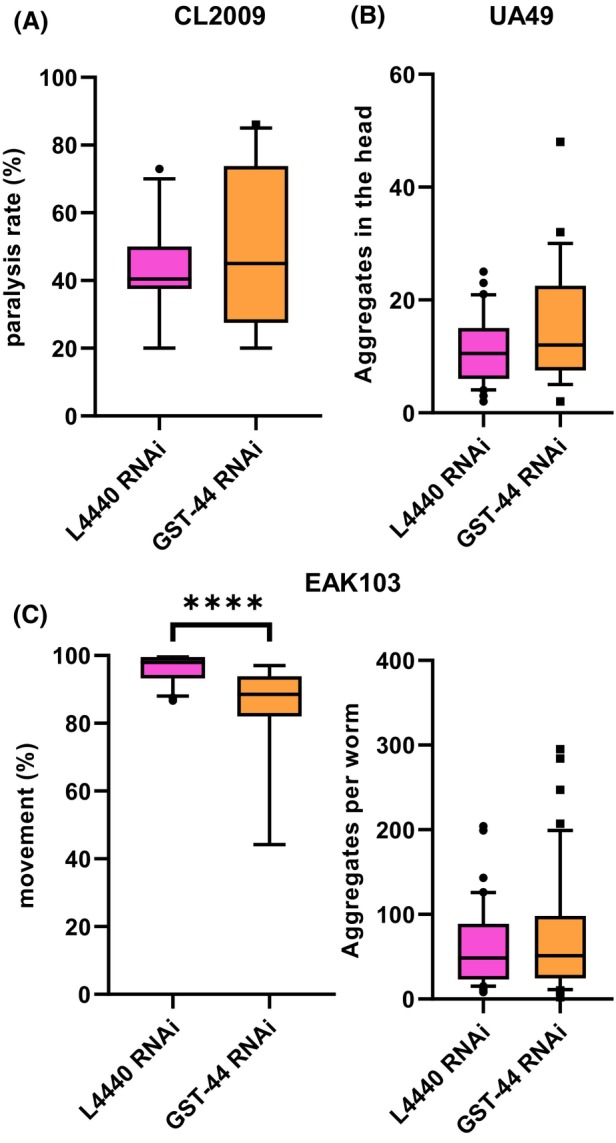
Analysis of the protective function of GST‐44 in the *C. elegans* models of neurodegeneration. (A) Following heat shock, the paralysis rate of strain CL2006 (Alzheimer's disease model) was measured. No significant differences were observed between gst‐44 knockdown and the control, indicating no specific protective role of gst‐44 in this model. (L4440 RNAi, *n* = 140; *gst‐44* RNAi, *n* = 100; *t*‐test). (B) Formation of alpha‐synuclein in *C. elegans* (Parkinson's disease model) was analyzed. GFP aggregates in the head region were counted and compared between gst‐44 knockdown and control. No significant differences were observed. (L4440 RNAi, *n* = 31; *gst‐44* RNAi, *n* = 30; *t*‐test). (C) To analyze the influence of GST‐44 in the Huntington's disease model, strain EAK103 was used. Movement and aggregate formation in the muscles were compared between *gst‐44* knockdown and control. While the movement rate of knockdown worms was significantly reduced (L4440 RNAi, *n* = 48; *gst‐44* RNAi, *n* = 48; *t*‐test), no statistical difference was observed for aggregate formation. (L4440 RNAi, *n* = 43; *gst‐44* RNAi, *n* = 46; *t*‐test).

Given that polymorphic variants of human GSTOs are associated with neurodegenerative disorders [25], we investigated the effect of GST‐44 knockdown in *C. elegans* models for Alzheimer's disease (CL2006), Parkinson's disease (UA49), and Huntington's disease (EAK103). In the CL2006 strain, which expresses the insoluble amyloid β‐peptide (Aβ_3–42_) leading to progressive paralysis in the body wall muscles, GST‐44 knockdown did not significantly alter the Aβ‐induced paralysis compared with the control treatment (bacteria containing the empty RNAi vector) (Fig. 7A). This was also the case in UA49 worms that were scored for α‐synuclein (α‐syn) aggregate size and number in body wall muscles (Fig. 7B). In the EAK103 strain, which expresses polyglutamin repeats (polyQ128) fused to yellow fluorescent protein under the unc‐54 promoter, resulting in protein aggregation and motility impairment, GST‐44 RNAi was also assessed for its effect. Although there was no significant change in protein aggregate levels, we observed a slight reduction in motility in the GST‐44 knockdown worms compared to controls (Fig. 7C).

### Phenotypic analysis of GST‐44 deletion mutants

**Fig. 8:**
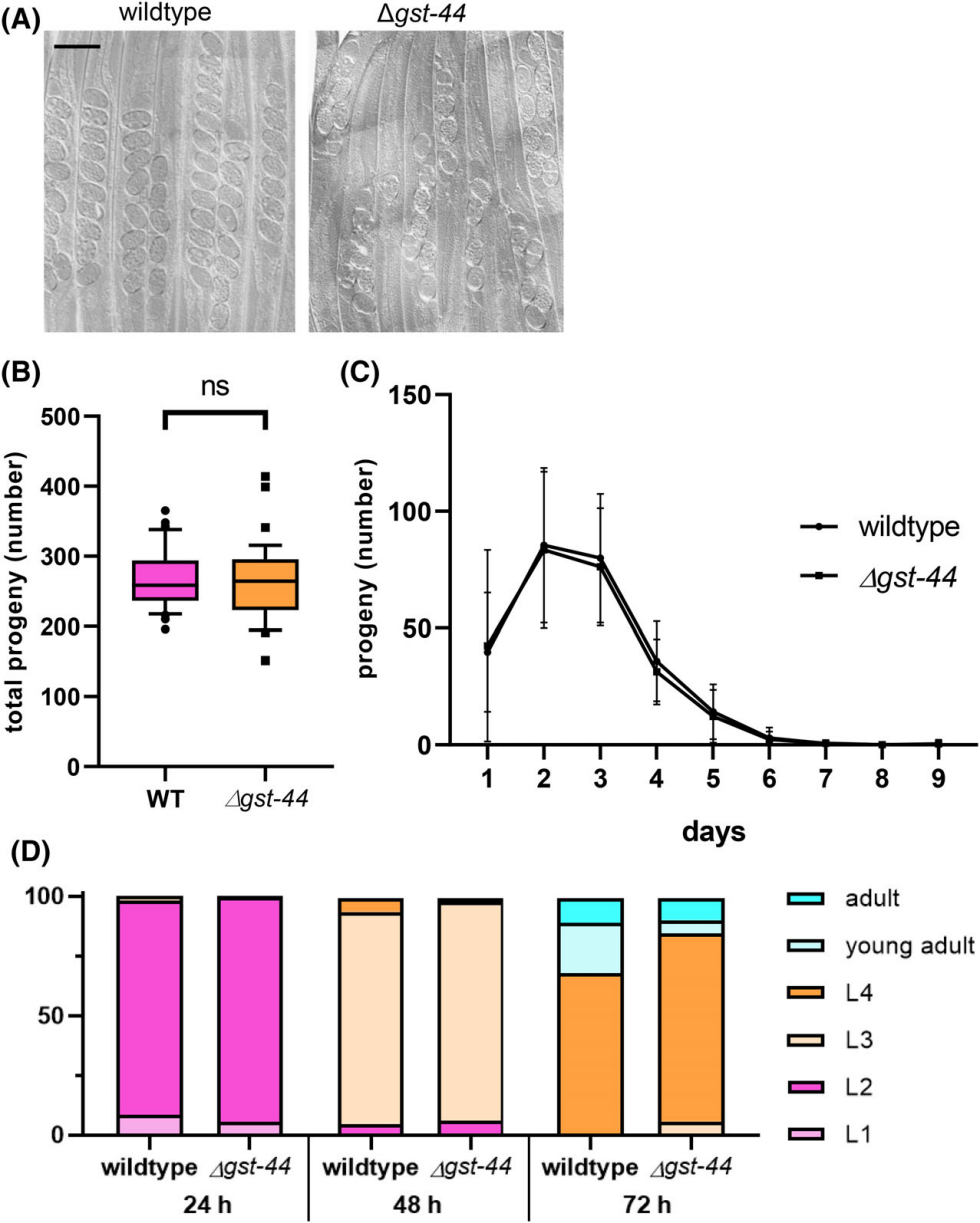
Life history traits of GST‐44 deletion mutants. (A) Gonad structure and egg arrangement were compared between wildtype and *gst‐44* deletion mutants. No discernible morphological differences were observed in gonads. However, in *gst‐44* deletion mutants, eggs showed irregular positioning and abnormal clustering compared to the linear arrangement in wildtype. Synchronized adult worms were paralyzed using levamisole and fixed on an agar pad. Scale bar: 100 μm (wildtype, *n* = 100; gst‐44, *n* = 100). (B, C) The reproduction rate and larval development of wildtype worms and gst‐44 deletion mutants were compared. No significant differences were observed in the total number of progeny (B) or the number of progeny per day (C) between the two groups (wildtype, *n* = 45; gst‐44, *n* = 38, *t*‐test). (D) Larval development was analyzed over 72 h, with no significant difference noted between wildtype and gst‐44 deletion mutants (wildtype, *n* = 147; gst‐44, *n* = 156, *t*‐test).

To gain further insights into the role of GST‐44 in *C. elegans*, the deletion mutant GST‐44(tm6133) V was examined. Here, no discernible morphological differences were observed. Interestingly, while in wild‐type worms fertilized eggs are typically arranged in a linear row as they move toward the uterus, a defect in the transit through the proximal gonad or uterus can be observed in the deletion mutant, leading to irregular positioning and abnormal clustering of eggs as they fail to align properly (Fig. 8A). Despite these irregularities in egg positioning, a closer examination of life history traits reveals that the GST‐44 deletion mutants maintain comparable reproductive output to wild‐type worms.

**Fig. 9:**
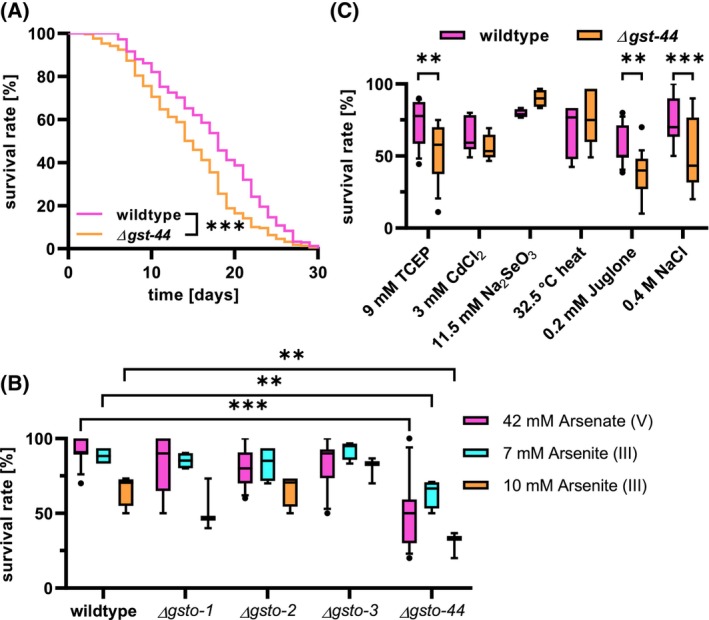
Analysis of *∆gst‐44* deletion mutant life span and survival under various stress conditions. (A) Lifespan analysis of the *∆gst‐44* deletion mutant compared to the wild type. Survival over time was significantly reduced for the *∆gst‐44* deletion mutant (*n* = 300 worms, average lifespan = 15 days) compared to the wild type (*n* = 300 worms, average lifespan = 18 days). Survival curves were generated from three independent experiments, with 100 worms per experiment. Significant differences are marked with *** for *P* < 0.001 (log‐rank test). (B) Survival rates of *C. elegans* omega‐class GST deletion mutants under As^V^ and arsenic stress were assessed. The omega‐class GST deletion mutants *∆gst‐1*, *∆gst‐2*, and *∆gst‐3* had survival rates comparable to the wild type, whereas *∆gst‐44* exhibited a strong reduction in survival rate. (C) Survival rate of the *∆gst‐44* deletion mutant and wild type following exposure to various stressors for 24 h. Survival is expressed as the percentage of worms alive after 24 h relative to the initial population (100% = all worms alive at the start of the experiment). Under TCEP, juglone, and NaCl stress conditions, the *∆gst‐44* deletion mutant exhibited a significant reduction in survival rate compared to the wild type. In contrast, no significant differences in survival were observed under cadmium, heat, or Na_2_SeO_3_ stress. For all conditions, *n* = 120 worms per group, except for TCEP (*n* = 160). Statistical significance was determined using Student's *t*‐test: ***P < 0.001, **P < 0.01.

When considering life history traits, no significant changes were observed in terms of reproduction or development (Fig. 8B–D). The GST‐44 deletion mutants laid 262.5 ± 55.4 eggs at 20 °C, while the wild‐type laid about 266.9 ± 40.7 eggs. However, the deletion mutant exhibited a highly significant reduction in lifespan compared to the wild‐type (Fig. 9A). On average, the deletion mutant had a lifespan reduction of 3 days when compared to the wild‐type, which had an average lifespan of about 18 days.

Next, the survival under different stress conditions was investigated, as omega‐class GSTs are frequently associated with stress‐related functions. Given that omega‐class GSTs have been shown to play a role in the metabolic conversion of As^V^ into As^III^ and its subsequent transformation into methylated metabolites [26], we analyzed both oxidation states. When exposed to both arsenic, a significant reduction in survival was evident when compared to the wild‐type. A dose‐dependent survival rate was observed with As^III^, with a reduction in survival of 26% at 7 mm and 36% at 10 mm As^III^. A 41% reduction of survival was observed with 42 mm As^V^. While the GST‐44 showed a significantly reduced survival rate under arsenic, this was not observed in the deletion mutants *gsto‐1*(eva301) III, *gsto‐2*(eva302) III, and *gsto‐3*(eva303) III (Fig. 9B).

Regarding the other stressors, a 25% reduction in the survival rate was observed when exposing the GST‐44 deletion mutants to the naphtoquinone juglone (0.2 mm), which induces oxidative stress by generating superoxide [27]. Additionally, the trialkylphosphine tris(2‐carboxyethyl)phosphine (TCEP), known to induce reductive stress in the ER lumen, reduced the survival rate of the deletion mutant by approximately 20% (Fig. 9C).

## Discussion

The phylogenetic tree, along with the alignment of protein sequences, reveals that GST‐44 is associated with the omega subclass of GSTs. In *C. elegans*, there are five genes encoding GSTOs, one of which has recently been reclassified from a pseudogene to a functional gene. Among these genes, only GSTO‐1 has been extensively characterized [18]. The functions of the other genes remain unknown. This study aimed to elucidate the role of GST‐44 in *C. elegans*.

Omega‐class GSTs exhibit varied enzymatic activities across species [28]. Due to a cysteine at the active center, GSTOs do not demonstrate activity with typical GST substrates such as CDNB or ethacrynic acid but rather exhibit activity with substrates typically associated with glutaredoxins or thioredoxins [4, 29]. In comparison to GSTO‐1 from *C. elegans* [18], GST‐44 displayed higher thioltransferase activity; however, the high K_M_ value obtained indicates the enzyme's low affinity for the substrate HEDS. Notably, GST‐44 exhibited tenfold higher glutathione peroxidase activity toward cumene hydroperoxide than the activity observed for GSTO‐1. Nevertheless, this activity is comparable to that of GSTO1 in *Apis cerana cerana* [30] or bmGSTO in the silkmoth [31]. Additionally, the disk diffusion assay illustrated the protective effects of GST‐44 against the oxidative stress inducer cumene hydroperoxide. *E. coli* strains overexpressing GST‐44 displayed enhanced resistance to prolonged oxidative stress. Similar results were observed for GSTO‐1 from *C. elegans* [18] and GSTO1 from *A. cerana cerana* [30].

The arsenate reductase activity of GST‐44 in *C. elegans* indicates a potential involvement in the metabolism and detoxification of arsenic. This activity reduces arsenate to arsenite, which then forms complexes with GSH. The formation of As‐GSH complexes is essential for the efflux of arsenicals from cells, as these complexes are preferred substrates for ATP‐binding cassette (ABC) transporters, facilitating the export of arsenicals and contributing to detoxification [32].

Our examination of the reporter strain OH2204 indicates that GST‐44 is primarily, yet moderately, expressed in the cytoplasm and nuclei of the anterior and posterior intestine as well as in the excretory system. In comparison, the GSTO‐1 [18] and GSTO‐2 are both expressed in the intestine, while GSTO‐3 is highly expressed in pharyngeal and body‐wall muscles (unpublished data). GST‐44 expression was unaffected by osmotic stress, cadmium, or rapamycin. However, GFP fluorescence increased after exposure to the fungal mold *P. brevicompactum*, suggesting the involvement of *GST‐44* as part of *C. elegans*' defense mechanisms against fungi in its natural habitat. Furthermore, exposure to the cyanobacteria strain *PCC 7806 Mut*, which primarily contains trypsin inhibitors, led to a strong upregulation of GST‐44 expression. Interestingly, no such upregulation was observed following exposure to *NIVA Cya 42*, a strain that predominantly contains chymotrypsin inhibitors.

Cyanobacteria harbor toxins as well as numerous other biologically active secondary metabolites. Among these, protease inhibitors are prevalent, with many targeting serine proteases such as chymotrypsin and trypsin [33]. The observed differential response may arise from the disruption of digestive processes by the trypsin inhibitor, which affects protein degradation or nutrient absorption, thereby inducing a stress response that triggers GST‐44 expression, unlike the chymotrypsin inhibitor. Additionally, the trypsin inhibitor may activate specific stress‐response signaling pathways not influenced by the chymotrypsin inhibitor, further leading to the upregulation of GST‐44 expression.

DMF belongs to the fumaric acid esters and functions as an activator of the transcription factor NRF2 [34]. DMF disrupts the interaction of NRF2 and its cytosolic regulator KEAP1 (Kelch‐like ECH‐associated protein 1), causing NRF2 to translocate to the nucleus and initiate a stress response. KEAP1's redox sensor function is known to involve multiple reactive cysteines that are modified by DMF [22]. Since NRF2 regulates the expression of cytoprotective, antioxidant, and anti‐inflammatory genes, DMF is used to treat multiple sclerosis and psoriasis [35].

Our study demonstrates that DMF exerts a similar impact on the NRF2 ortholog SKN‐1, leading to strong induction of the representative target gene *gst‐4* in *C. elegans*, despite the absence of KEAP1 in its genome. As suggested by Choe *et al*., the function of KEAP1 could be taken over by the WD40‐repeat protein WDR‐23 that also possesses multiple cysteines, four of which are particularly sensitive to electrophilic influence due to their surrounding amino acids, and thus could function as a redox sensor [36].

Additionally, our findings using GST‐44::GFP worms treated with DMF and subjected to SKN‐1 knockdown indicate that GST‐44 activation is indeed mediated by SKN‐1 induction rather than changes in cellular redox status influenced by DMF, as noted in previous studies [22].

After establishing GST‐44 as a target gene for SKN‐1, we were interested in exploring the potential involvement of other transcription factors in regulating GST‐44 under arsenic stress. Since the transcription factor PHA‐4 has already been shown to be involved in the response to arsenic [37], the activation of GST‐44 by PHA‐4 in response to arsenic comes as no surprise. *Pha‐4* that encodes a forkhead box (FOX) A transcription factor homolog, not only has a function in pharynx development but also in caloric restriction longevity [38] and regulation of genes related to xenobiotic metabolism [39].

FOXA has been identified as an initial and important chromatin binding factor that binds to the genome for a period prior to transcriptional activation and before other transcription factors. The ability to bind to its target sites, even in condensed chromatin, has also been shown for PHA‐4 [40]. Interestingly, in fumarate hydratase deficient cells that have a high accumulation of intracellular fumarate and thus a persistent oxidative stress environment, the forkhead transcription factor FOXA2 was identified to directly regulate the antioxidant response coordinated by NRF2 [41].

In *Drosophila*, the overexpression of GSTO‐1 has been shown to effectively rescue neurodegenerative phenotypes by reducing neuronal damage and improving motor function [42]. Conversely, our investigations in *C. elegans* indicate that GST‐44 does not confer any protective effects. Notably, GSTO‐3 exerts a significant influence in the *C. elegans* UA49 Parkinson's model, markedly attenuating disease progression (data not shown). These results highlight the distinct functional roles of Omega‐class GSTs in *C. elegans*, where GST‐44 fails to provide neuroprotection, whereas GSTO‐3 demonstrates a pronounced protective effect in neurodegenerative contexts.

Genetic manipulations of stress‐responsive proteins can have diverse effects on the lifespan of model organisms. In the case of *C. elegans*, the absence of GST‐44 significantly shortened the lifespan. Conversely, when GSTO‐1 is knocked down, it does not result in a reduced lifespan for the mutant worm [18]. This could be linked to the residual activity that remains after RNAi‐induced knockdown or the compensatory capacity of the highly homologous GSTO‐2 for the loss of GSTO‐1. Given the absence of observable effects on reproduction or development, it is likely that GST‐44 is not primarily involved in general fitness but rather in stress response. This supposition is reinforced by notably lower survival rates of the GST‐44 deletion mutant under various stress conditions.

The protective function of GSTs against the naturally occurring mitochondrial toxin juglone has been demonstrated for various GSTs in both parasitic and free‐living nematodes [18, 43, 44, 45]. Exposure of *C. elegans* to juglone results in increased ROS levels and the formation of glutathione adducts. This process leads to glutathione depletion and activates the SKN‐1‐dependent antioxidant response, which in turn induces GST‐44. On the other hand, when GST‐44 is absent, juglone treatment becomes much more toxic. Similarly, the reducing agent TCEP inhibits disulfide bond formation, causing proteins to be unfolded or misfolded [46]. This leads to ER stress and the accumulation of misfolded or impaired proteins, which likely explains why the survival rate is lower in the *gst‐44* deletion mutant.

In *C. elegans*, we demonstrate that As^III^ exhibits higher toxicity compared to As^V^, consistent with comparative *in vitro* studies conducted on human cell lines, which indicated that a lower oxidation number correlates with greater toxicity [47]. The central aspect of the interaction between GSH and arsenic lies in its function as a reductant, facilitating the conversion of As^V^ to its trivalent form. Subsequently, the resulting As^III^ species rapidly form complexes with GSH, establishing an integral relationship between GSH‐mediated reduction and complexation processes within cellular systems [48].

In comparison with the other omega‐class deletion mutants, the reduced survival of the GST‐44 deletion mutants under arsenic stress indicates its potential involvement in arsenic metabolism. Arsenic's toxic effects involve accumulation of the metalloid in the cell, mutations due to inhibited DNA repair, interference with the active site cysteines of proteins, and the generation of reactive oxygen species [49]. As a result, arsenic is not only directly harmful itself but also induces additional oxidative stress.

In a previous study, we conducted an RNAi‐screen, targeting GSH‐related genes to investigate survival rates under sublethal concentrations of various stressors [50]. Our findings distinctly revealed the critical role of GSH synthesis, particularly of γ‐glutamylcysteine synthetase, in protecting against As^III^. Though with a somewhat subtler effect, the alpha‐class GST‐32, an extracellular glutathione peroxidase (C11E4.1) and the GST‐44 impacted survival rates under As^III^ stress. Both GST‐32 and GPX are evidently involved in combating oxidative stress through their GSH‐dependent reduction of peroxides or by eliminating byproducts of lipid peroxidation. This holds true for GST‐44 as well.

The importance of GST‐44 in the response to arsenic is further underscored by its substantial upregulation upon exposure to arsenic. While a comparable upregulation of GST‐44 in the intestine is observed upon exposure to the oxidative stressor Juglone, it is noteworthy that under arsenic stress conditions a pronounced upregulation can be specifically observed in the worm's excretory system. This implies that the excretory system may have a vital role in arsenic metabolism. Given that the excretory system is believed to function similarly to a renal system, contributing to the maintenance of osmotic balance and waste elimination [51], its involvement in arsenic detoxification appears plausible.

Both human omega‐class GSTs have been implicated in arsenic biotransformation, where the methylarsonate reductase is identical to the human GSTO‐1. The human GSTO‐1 was also shown to catalyze the reduction of inorganic arsenic [5], a function that we also observe with the GST‐44. The resulting end product of human arsenic biomethylation, dimethylarsinic acid, is rapidly excreted through the renal system [52, 53]. While biotransformation through methylation has been considered the primary mechanism for arsenic detoxification in mammals, the absence of an arsenic methyltransferase homolog in the *C. elegans* genome, as noted by Thomas *et al*., suggests that *C. elegans* lacks the ability to methylate inorganic arsenic, indicating the presence of alternative detoxification mechanisms for inorganic arsenic [54].

In conclusion, our study highlights the pivotal role of GST‐44 in *C. elegans* for arsenic detoxification and overall stress response. The arsenate reductase activity of GST‐44, along with its differential tissue expression and regulatory interactions with key transcription factors SKN‐1/Nrf2 and PHA‐4 underline its multifaceted function. Although GST‐44 does not exhibit neuroprotective properties as seen for GSTO‐3, its involvement in lifespan and stress resilience suggests broader biological implications. Altogether, these insights advance our understanding of GST‐44's essential contributions to stress adaptation and defense mechanisms in *C. elegans*.

## Material and methods

### Database search and bioinformatics analysis

The evolutionary history of *C. elegans* GSTs was inferred using the Minimum Evolution (ME) method [55]. The evolutionary distances were computed using the Poisson correction [56] and are in the units of the number of amino acid substitutions per site. The ME tree was searched using the Close‐Neighbor‐Interchange algorithm [57] at a search level of 1. The neighbor‐joining algorithm [58] was used to generate the initial tree. This analysis involved 50 amino acid sequences. All ambiguous positions were removed for each sequence pair (pairwise deletion option). Evolutionary analyses were conducted in MEGA X [59].

Further analyses of the *gst‐44 and* the promoter region were conducted using wormbase (wormbase.org, WS282), signalP5 [60], ConTra v3 [61] and InterPro [62].

### Cloning, expression, and purification of the recombinant protein

GST‐44 was amplified via PCR from *C. elegans* cDNA using the oligonucleotide primers gst‐44 s and gst‐44as (Table 1, Eurofins Genomics GmbH, Hamburg, Germany). The resulting PCR product was then cloned into the expression vector pJC40, which features a removable N‐terminal histidine tag and enables purification through metal chelate chromatography [63].

**Table 1:** Primers used in this study.

Primer	Sequence 5′ → 3′	Temp. (°C)
Primers used for expression in pJC40		
gst‐44 pJC40 S	GCGC**CATATG**TCGGTGCTCGAAGGAATC	69.5
gst‐44 pJC40 AS	GCGC**GGATCC**TTACAAGCCATAATCAAA	65
Mutagenesis primer		
gst‐44 C32G_mut_S	CATTTATAGCATGCGCTTCGGCCCTGCTGCTCAGCG	70
gst‐44 C32_mut_AS	CGCTGAGCAGCAGGGCCGAAGCGCATGCTATAAATG	70
Sequencing primers for control of deletion mutants		
Del gst‐44 S	GAATGTCGGTGCTCGAAGGA	59.4
Del gst‐44 AS	GACAGTTGGGCTTCCAGAATG	60
CRISPR/Cas9 of GSTOs		
gsto‐1#6 fwd	TCTTGAAGATCCCTTTGATAGCGG	61
gsto‐1#6 rev	AAACCCGCTATCAAAGGGATCTTC	61
gsto‐2#2 fwd	TCTTGACAATATGCGATATTGCCCA	60
gsto‐2#2 rev	AAACTGGGCAATATCGCATATTGTC	60
Repair template gsto‐2	CCAGCTTCAGGAACCATTCGTATCTACAATATGCGATATTGCCCAGCGTAGGTAGGTAGGATCCGCGTGGGCTCAACGTGCTCTAATCTTTGCGTCTC	
gsto‐3#1 fwd	TCTTGCTACACTTGCTAGTTGGGTG	63
gsto‐3#1 rev	AAACCACCCAACTAGCAAGTGTAGC	63
Repair template gsto‐3	CCTACCTGGGAATTGTTCCACGTCGGCGGCACTATGTATGCAATTTCTGCGTAGGTAGGTAGGATCCGCGCACCCAACTAGCAAGTGTAGCAATTGATCAGGCTTATCAGGTACG	

Bold values indicate restriction enzyme cutting sites.

Overnight cultures of the *E. coli* strain BL21(DE3) expressing GST‐44 were used to inoculate 0.5 L cultures in LB medium with ampicillin (Applichem, Darmstadt, Germany). The liquid cultures were incubated at 130 rpm and 37 °C to an OD_600_ of 0.5 to 0.8. Expression of the GST‐44 was induced by the addition of isopropyl β‐d‐1‐thiogalactopyranoside (IPTG) (1 mm, final concentration; Applichem) and incubated for another three hours. The cells were harvested by centrifugation (20 min at 6000 ***g*** and 4 °C). The supernatant was discarded, and the pellets were frozen at −20 °C or used for further processing.

Cell lysis was performed by sonication, and protein purification was accomplished using Ni‐NTA agarose according to the manufacturer's protocol (Macherey‐Nagel, Düren, Germany). Protein concentration was determined by the method of Bradford [64].

The homogeneity of the enzyme preparation was analyzed by 12.5% SDS/PAGE. Proteins were revealed by Coomassie Blue staining and western blot analysis using a mouse monoclonal antibody against the 6 × his‐tag (Dianova, Hamburg, Germany) at a dilution of 1:1000. A monoclonal anti‐mouse antibody from donkey with alkaline phosphatase (Dianova) was used as a second antibody at a dilution of 1:10 000. For some of the enzymatic activity tests, a buffer change was necessary. The elution fractions were combined and incubated overnight at 4 °C under constant stirring in a dialysis tube to replace the elution buffer with PBS buffer.

### Disk diffusion assay

The impact of heterologous expression of GST‐44 in *E. coli* cells under oxidative stress conditions was evaluated using a disk diffusion assay. For this purpose, 3 mL of top agar, composed of 50% LB media, 50% LB agar, 0.5 mm ampicillin and 1 mm IPTG, was prepared and mixed with 200 μL of an overnight culture of the GST‐44 overexpressing *E. coli* strain BL21(DE3) or a control strain carrying an empty pJC40 vector. The resulting mixture was spread onto LB‐ampicillin agar plates and allowed to dry briefly. Subsequently, filter disks were placed on top of the agar and saturated with 3 μL of various concentrations of cumene hydroperoxide (Sigma Aldrich Chemie GmbH, Taufkirchen, Germany), a commonly used oxidative stressor. Furthermore, disk diffusion assays with 500 mm As^III^ (Sigma Aldrich Chemie GmbH) were performed as well. The compounds diffused from the filter disks into the surrounding agar, affecting the bacterial growth. The plates were then incubated overnight at 37 °C and the resulting inhibition zones were measured. Statistical analysis was performed using the Student's *t*‐test to determine any significant differences.

### Enzymatic activity assays

To further define the properties of GST‐44, a series of enzymatic activity assays were performed. First, the GSH‐conjugating activity was assessed using the generic GST substrates CDNB (Sigma Aldrich Chemie GmbH) [65] and ethacrynic acid (Sigma Aldrich Chemie GmbH) [66]. To evaluate the glutathione peroxidase activity of GST‐44, cumene hydroperoxide was employed as a substrate [67]. The thioltransferase activity of GST‐44 was assessed using the substrate HEDS (Sigma Aldrich Chemie GmbH) [68]. This assay aimed to examine the enzyme's ability to catalyze the reduction of spontaneously produced 2‐mercaptoethanol. DHAR activity was investigated by monitoring the formation of ascorbic acid [69]. The assessment of activity toward MMA^V^ (Santa Cruz Biotechnology Inc., Heidelberg, Germany) involved monitoring the oxidation of GSH (Sigma Aldrich Chemie GmbH) by measuring the reduction of the generated GSSG using GSH reductase and determining the concomitant NADPH (Applichem) oxidation [70].

All enzymatic activity assays were performed in triplicates using three independent enzyme preparations. Additionally, *K*_m_ values for the HEDS substrate were determined using a substrate saturation curve and a Lineweaver–Burk diagram.

### *Caenorhabditis elegans* strains and cultivation

The *C. elegans* wild‐type (N2 Bristol) and the GFP reporter strains OH2204, *otEx1182 [gst‐44p::gst‐44::gfp + rol‐6(su1006)]*, CL2166, *dvIs19 [gst‐4p::GFP::NLS]* and LD1008, *ldEx9 [skn‐1(operon)::gfp + rol‐6(su1006)]* were obtained from the *Caenorhabditis* Genetics Centre (Minneapolis, MN, USA). The deletion mutant strain *gst‐44(tm6133)* was obtained from the National Bioresource Project (NRBP), Tokyo (Japan). This strain contained a deletion starting at amino acid position 91 and was outcrossed with N2 wild‐type six times before use.

For CRISPR/Cas9‐mediated genome editing of GSTO‐1(WWU301 *gsto‐1*(eva301) III), WWU302 *gsto‐2*(eva302) III, WWU303 *gsto‐3*(eva303) III, we used *dpy‐10(cn64)* as the co‐CRISPR marker [71] and pJW1285 (Addgene Inc., Watertown, MA, USA) to express both guide‐RNA (gRNA) and Cas9 enzyme [72]. Following microinjection, F1 generation *dpy‐10*(cn64) rollers were screened for co‐edits via PCR, verified by Sanger sequencing followed by outcrossing (list of PCR primers, gRNA and ssOligo donor sequences, Table 1).

*Celegans* was cultured on nematode growth media (NGM) plates seeded with *E. coli* OP50 at 20 °C according to standard conditions [73]. Synchronization of the worms was achieved by hypochlorite lysis [74].

### RNAi treatments

RNAi was carried out following standard procedures [75]. RNAi treatments were performed by feeding *C. elegans* HT115 *E. coli* carrying RNAi clones in the pL4440 vector. Synchronized L1 were transferred to seeded plates containing ampicillin and IPTG, which produce the dsRNA for the desired knockdown or an empty control plasmid.

### Gene expression analysis under normal and stress conditions

To assess the expression pattern of GST‐44, the GFP reporter strain OH2204 was utilized. Following L1 synchronization, worms were propagated on NGM agar plates seeded with OP50. For the larval stages, images of worms were captured using the laser‐scanning microscope LSM 510 META (Carl Zeiss Microscopy GmbH, Jena, Germany) every 24 h for 3 days.

To investigate the induction of the GFP reporter strain OH2204 under varying stress conditions, assays were performed with staged L4 or young adults, and GFP signals were quantified under normal and short‐term stress conditions. Stressors were applied under the following conditions: 10 mm sodium arsenic (4 h) and 50 μm rapamycin (4 h, Applichem) in liquid (M9 buffer) and 0.2 mm Juglone (4 h, Sigma Aldrich Chemie GmbH), 8.2 mm cadmium chloride (4 h, Sigma Aldrich Chemie GmbH) and 400 mm NaCl (4 h, Applichem) on NGM agar plates. All stressors were used at the specified final concentrations. Notably, OP50 bacteria were not included during the exposure period to avoid interference with the stress condition.

Since induction of xenobiotic‐metabolizing enzymes has frequently been observed in response to exposure to pathogens, the GFP reporter strain OH2204 was exposed to the fungi *Penicillium brevicompactum* Dierckx (obtained from the Leibniz Institute DSMZ‐German Collection of Microorganisms and Cell Cultures) following the procedure by Wallace *et al*. [76], *Serratia marcescens* strain HY, and the *Microcystis aeruginosa* strains NIVA Cya 43 [77] and PCC 7806 Mut [78] containing either chymotrypsin or trypsin inhibitors but no microcystins.

For imaging, worms were placed on a slide with a drop of 1% agarose (Carl Roth GmbH, Karlsruhe, Germany) and immobilized using levamisole (100 μm; Sigma Aldrich Chemie GmbH). For each test condition, four independent assays were conducted. For data evaluation, Leica Application Suite 2.6.0 software was used. Image quantification was carried out using the imagej software.

### Dimethyl fumarate treatment

To investigate whether DMF (Sigma Aldrich Chemie GmbH), a known pharmacological activator of Nrf2 (NF‐E2‐related factor 2) is able to stimulate Skn‐1 translocation into the nucleus and induce the transcription of GST‐44, DMF was dissolved in dimethyl sulfoxide (DMSO; Applichem) at a concentration of 200 mm. Small plates were then prepared with a final concentration of 2 mm DMF. As a control, plates with an adjusted DMSO concentration were used. After incubation for 18 h, the images were acquired as previously described.

### *C. elegans* models of neurodegenerative diseases

Worm strains expressing Aβ (CL2006, *dvIs2*[pCL12(P_*unc–54*_::Aβ), pRF4]), α‐syn (UA49, *baIn2*[P_unc–54_::α‐syn::GFP, rol‐6 (su1006)]) or a fragment of mutant human Huntington protein (EAK103, *eeeIs2*[unc‐54p::Htt513(Q128)::YFP::unc‐45 3′UTR]) were scored for neurodegeneration following RNAi of GST‐44.

Paralysis of CL2006 is caused by Aβ expression and accumulation in *C. elegans* body wall muscle. Worms were kept at a temperature of 16 °C. To speed up paralysis, worms were cultured at 37 °C for 1 h. Worms were considered paralyzed if they displayed “halos” of cleared bacteria around their heads, moved only their heads, or did not respond at all when gently touched with a worm pick.

UA49 animals were scored for aggregate size and number 2–3 days post hatch according to Starr *et al*. [79].

EAK103 expresses a YFP‐tagged polyQ‐expanded disease‐associated 513 amino acid fragment of human Htt in body wall muscle cells.

### Lifespan and stress resistance assays

To determine the lifespan, synchronized worms were grown on NGM plates until they reached the L4 larval stage. Ten worms were picked on one small plate and moved to a new plate every day while counting live or dead animals (*n* = 300). The survival was measured each day via touch response. Significant differences were calculated with the Kaplan–Meier Method using a Log‐Rank test.

For brood size determination, L4 individuals were placed on a small plate and transferred to a new plate every 24 h for nine to 10 days. Hatched offspring were counted on the following day. Additionally, developmental stages were evaluated at 24, 48, and 72 h after egg laying. Here, 3–5 adult worms were placed on a small plate and removed after laying approximately 10–15 eggs (*n* = 120). The subsequent development of these eggs was observed over a period of 3 days.

For stress survival assays, age‐synchronized populations of young adult worms were utilized. They were placed on small *E. coli* seeded NGM plates containing stressors such as the ER stress inducing agent TCEP (Carl Roth GmbH, Karlsruhe, Germany), cadmium chloride (Sigma Aldrich Chemie GmbH), natrium selenite (Sigma Aldrich Chemie GmbH), the redox quinone juglone (Sigma Aldrich Chemie GmbH), and NaCl (Applichem) on NGM agar plates. As^III^ or As^V^ (Applichem) exposure was performed with liquid media under shaking at 20 °C [50, 80]. After 18 h or 24 h, respectively, survival of the worms was checked by touch stimulus. All experiments were performed independently at least three times. Statistical differences were calculated using a Student's *t*‐test.

## Conflict of interest

The authors declare no conflict of interest.

## Author contributions

ML, DL, LR, SM, and RP performed the research. ML wrote the draft and edited the final version. CSK and ML supervised all experimental procedures. ES and MN critical review and editing. AM‐V and EL contributed conception and design of the work. EL wrote the final version. All authors read and approved the final manuscript.

## Data Availability

The data that support the findings of this study are available from the corresponding author [liebaue@uni-muenster.de] upon reasonable request.
